# Opportunities Offered by Telemedicine in the Care of Patients Affected by Fractures and Critical Issues: A Narrative Review

**DOI:** 10.3390/jcm14207135

**Published:** 2025-10-10

**Authors:** Giulia Vita, Valerio Massimo Magro, Andrea Sorbino, Concetta Ljoka, Nicola Manocchio, Calogero Foti

**Affiliations:** 1 Physical and Rehabilitation Medicine, Clinical Sciences and Translational Medicine Department, Tor Vergata University, 00133 Rome, Italy; giulia.vita@students.uniroma2.eu (G.V.); valeriomassimo.magro@students.uniroma2.eu (V.M.M.); andrea.sorbino@ptvonline.it (A.S.); concetta.ljoka@ptvonline.it (C.L.); 2 PhD in Tissue Engineering and Remodeling Biotechnologies for Body Function, Clinical Sciences and Translational Medicine Department, Tor Vergata University, 00133 Rome, Italy

**Keywords:** bone fracture, mobile applications, rehabilitation, quality of life, telemedicine, telerehabilitation

## Abstract

Telerehabilitation is an effective, accessible addition or alternative to conventional rehabilitation for fracture management, especially in older adults after hip fractures, leveraging video visits, mHealth apps, virtual reality (VR), and wearable sensors to deliver exercise, education, and monitoring at home with high satisfaction and adherence. Across non-surgical and surgical contexts, telemedicine shows feasibility and cost benefits, with mixed superiority but consistent non-inferiority for functional outcomes versus in-person care. In hip fracture populations, randomized and non-randomized studies indicate improvements in functional independence measure (FIM), Timed Up and Go test (TUG), Activities of Daily Living/Instrumental Activities of Daily Living (ADLs/IADLs), and quality of life, with some evidence for reduced anxiety and depression, while effects on mobility, pain, and adverse events remain uncertain overall. In patients with upper-limb fractures, telerehabilitation appears to improve function and pain, though strength gains may lag compared with in-person therapy in some trials; adjuncts like motor imagery and virtual reality may enhance outcomes and motivation. Application is facilitated by user-friendly platforms, caregiver involvement, and simple modalities such as structured phone follow-up. Limitations include small samples, heterogeneous protocols, scarce long-term data, and a predominance of non-inferiority or complementary designs, warranting larger, definitive trials. This technology can lead to improved patient management at home, effortlessly verifying treatment compliance, efficacy, and safety, while simultaneously reducing the need for hospitalization, promoting a more peaceful recovery. Here, we have undertaken a narrative review of the medical–scientific literature in this field.

## 1. Introduction

Increased life expectancy has led to an epidemiological shift in major chronic and degenerative diseases, with both their prevalence and incidence rising [1,2]. This increased prevalence and incidence has especially affected individuals over the age of 65, who are nowadays characterized by a greater degree of frailty than in previous periods [3,4]. This phenomenon has affected the whole Western world, and particularly countries with a significant proportion of elderly populations (e.g., Italy). Hip, femoral, and spinal fractures are examples of the consequences of this frailty and are an increasingly common occurrence among older adults in Italy [5,6,7].

Given the aging population, the global number of hip fractures is projected to increase from 1.26 million in 1990 to 4.5 million by 2050. The direct costs associated with this condition are and will be enormous, as the condition requires prolonged hospitalization and subsequent rehabilitation care [8,9]. Physicians specialized in Internal Medicine, Geriatrics, Orthopedics and Physical and Rehabilitation Medicine (PRM) are often consulted to evaluate patients with acute, subacute, or chronic manifestations and the outcomes of fractures of a different nature [10,11,12,13,14,15,16,17]. PRM specialists play an important role in the care of patients with fractures since therapeutic exercise and physical reconditioning have proved to be efficient in improving independence in Activities of Daily Living (ADL), cognitive functions, nutritional and physical conditions, balance, and muscle strength in the elderly with fractures. This should all be framed under a well-coordinated and designed Individual Rehabilitation Project (IRP) [18,19,20,21]. During the COVID-19 pandemic, healthcare costs further increased globally [22,23,24]. At the same time, the health crisis created during the pandemic, with the saturation of the healthcare system due to the number of hospitalizations of patients acutely suffering from severe respiratory illnesses, worsened the care of patients with fractures. Indeed, the difficult management of hospital beds and acute care in wards led, for example, to the deferral of fracture interventions well beyond the limits recommended by guidelines [25,26,27], to the undertreatment of osteoporotic and frail patients at risk of fracture, and to the prolongation of transfers to rehabilitation clinics, resulting in significant overall difficulties in properly managing resources and processes for these types of patients [28,29,30].

Nonetheless, during the COVID-19 pandemic, there was a renewed emphasis on rational resource allocation and their use to relaunch healthcare systems that prioritize hospitals for patients requiring high-intensity care and severe acute conditions. Community-based care and home care were emphasized as alternatives for patients with less intensive needs [31,32]. These observations led to the idea of leveraging existing and emerging technologies (simulation programs, artificial intelligence, computer networks, and management systems with teleconsultation capabilities) to support patient care remotely via so-called “telemedicine” [33]. Telemedicine has emerged as a transformative method for delivering healthcare services, particularly in rehabilitation (i.e., telerehabilitation), reflecting its multifaceted benefits for a diverse population. One of the primary advantages of telerehabilitation is its capacity to eliminate geographical barriers associated with traditional rehabilitation methods. Patients in rural and underserved areas can access rehabilitation services without the burden of extensive travel, thus increasing the overall accessibility of care [34,35]. This enhanced accessibility is particularly pertinent as it not only improves patient engagement but also enhances adherence to IRPs, resulting in improved physical function and quality of life over the long term [36,37]. Additionally, a systematic review by Wang et al. indicates that telerehabilitation not only saves time but also leads to higher patient satisfaction due to reduced waiting times and the convenience of engaging in therapy from home [38]. Telerehabilitation appears to significantly reduce healthcare costs for both patients and providers, mitigating the need for transportation and thereby reducing travel-related expenses [39]. Beyond convenience and cost-effectiveness, telerehabilitation includes the flexibility to customize therapeutic interventions based on individual patient needs. Healthcare professionals can utilize various digital formats, such as video conferencing and dedicated rehabilitation applications, to tailor exercises according to patient capability and progress [40,41]. The incorporation of telerehabilitation has proven instrumental during the COVID-19 pandemic, where direct access to healthcare services was significantly hindered. As face-to-face assessments posed risks, telerehabilitation provided a means to continue essential therapeutic interventions while maintaining safety protocols. Research indicates that providers and patients reported positive experiences and outcomes during these challenging times, thus establishing telerehabilitation as a viable and effective alternative in the realm of healthcare delivery [42].

Given the increased frailty and risk of fractures, with the resulting increase in healthcare costs, telerehabilitation appears to be an effective means of reducing these risks. The aim of this narrative review is to offer a comprehensive analysis of the existing literature on the possible application of telemedicine, and, in particular, telerehabilitation, as a new, innovative tool for fracture management.

## 2. Use of Telemedicine in Clinical Conditions

Telemedicine and telerehabilitation have already been applied effectively in non-surgical conditions (Table 1). An important example of the use of telemedicine, implemented after the COVID-19 pandemic, concerns diabetes. Telemedicine offers important opportunities, both in hospital and community settings, to monitor patients with diabetes [43,44]. Through dedicated software, physicians can communicate with patients at home, asking for clinical information, verifying adherence to treatment, and monitoring glycemic control (blood sugar, glycated hemoglobin, and ketones) [45,46,47]. Many studies have shown the benefits of this approach on glycemic control, both in type 1 and type 2 diabetes [48,49].

In addition to metabolic diseases, a recent trial on more than one hundred patients has verified the effects of telemedicine on patients suffering from respiratory diseases [50]. In terms of rehabilitation, a randomized controlled trial by Zanaboni et al. studied the remote supervision of patients with chronic obstructive pulmonary disease (COPD) followed for two years [51]. In a multicenter, single-blind, randomized, controlled clinical trial by Hansen et al., patients were assigned to either a 10-week telemedicine pulmonary re-educational treatment (60 min three times a week) or a conventional, non-telemedicine pulmonary re-educational treatment (90 min twice a week); results showed no superiority of remote treatment and supervision over conventional re-educational [52]. Another study on 65 patients by Cameron-Tucker et al. also showed no substantial benefit [53] and even a publication by Berkhof et al. did not report positive effects [54].

On the contrary, the group of Nagatomi et al. praised telemedicine as a tool to better follow fragile patients with heart failure, in a safer way and at their home [55]. Cardiac telemetry has long been used in cardiology departments and in rehabilitation units for patients suffering from heart failure and post-cardiac surgery. The expansion of the monitoring systems, placing them at a more considerable distance from the bed-monitoring stations in the open space of the department, offers several advantages. First, it enhances patient comfort. Second, in certain studies, such as the one conducted by Wita et al. [56] or in large trials (such as the BEAT-HF or the CardioBBEAT) [57,58], it has been demonstrated to reduce the likelihood of rehospitalizations for these patients. Regarding rehabilitation, prominent cardiology scientific societies, such as the European Society of Cardiology, have proposed the potential of telerehabilitation with patient supervision, ensuring the absence of adverse events. This underscores the safety of the protocols employed in this delicate patient group, while leaving the crucial task of precisely determining its clinical benefits to future trials [59].

In the oncology field, to reduce burdensome travel and provide remote or telephone monitoring of patients, new devices have been developed. The implementation of mobile technologies, particularly through dedicated apps, is a subject of numerous studies in the medical literature. For instance, the eSMART study demonstrated the effectiveness of remote symptom monitoring [60]. In a randomized trial conducted by Galiano-Castillo et al., a telemonitoring program for breast cancer patients was evaluated. The study involved 81 patients and found that the telemonitoring program was beneficial in assessing the outcomes of the patients followed and useful for monitoring rehabilitation exercises and their impact on quality of life [61].

Telemonitoring has also proven effective in detecting blood glucose levels continuously over 24 h [62], the presence of malignant arrhythmias [63], and remotely viewing spirometry tracings from the doctor’s office [64,65,66]. Additionally, it can be used to monitor electrocardiograms [67].

## 3. Use of Telemedicine in Surgical Conditions

Telemedicine and telerehabilitation have also been studied in patients undergoing surgical procedures (Table 2).

A randomized study conducted by Babar et al. compared patients who underwent in-person consultations with a specialist after surgery to those who received postoperative telematic monitoring by a urologist [68]. Shin et al. evaluated patients’ satisfaction with teleconsultation after undergoing pelvic floor reconstructive surgery [69]. In gynecological surgery, trials have explored the effects of telemedicine on monitoring patients’ disabling symptoms, including pain, sleep disturbances, stress, and other mental health disorders [70]. Additionally, they have investigated the impact of virtual visits compared to in-person consultations [71,72].

Bernason et al. conducted a clinical trial with cardiac surgery patients using telemonitoring to monitor their symptoms. The results were promising, with subjects returning to their preoperative levels of functioning within 3 to 6 months after coronary artery bypass grafting. Moreover, they increased their physical activity levels compared to their reported preoperative levels. Interestingly, cardiac rehabilitation is a class IA recommendation for patients with cardiovascular diseases [73]. Physical activity is the core component of a cardiac re-educational program [74]. However, many patients with cardiovascular diseases are failing to meet cardiac rehabilitation guidelines that recommend moderate-to-vigorous intensity physical activity. For this purpose, telerehabilitation interventions are effective at increasing minutes per week of moderate-to-vigorous intensity physical activity among patients in cardiac rehabilitation [75]. While existing studies in the literature have not demonstrated significant benefits in terms of outcomes, such as reduced anxiety, in patients undergoing aortic aneurysm surgery [76], remote telemonitoring of vital signs remains beneficial for early detection of red flags in these complex patients, even after surgery [77,78].

The positive impact of the telemedicine intervention is also described in thoracic surgery, in patients who have undergone lung transplantation [79,80], and in patients affected by lung cancer, treated surgically, also from a rehabilitation perspective [81,82].

## 4. Methods

The PubMed database was searched for clinical experiences and studies with the following key terms: “telerehabilitation,” “fractures,” in combination with the Boolean operator “AND”. The search was limited to studies on humans, encompassing all study types (e.g., case reports, clinical trials, reviews, and randomized controlled trials). The search was expanded through the bibliography of the retrieved texts. Considering this is a narrative review, a formal inclusion and exclusion criteria selection was not performed, but the search was maintained as broad as possible to achieve a comprehensive summary of current literature of the topic. Only papers written in English were considered.

Studies that did not mention a specific telerehabilitation methodology or that only talked about fractures from an orthopedic, geriatric or internistic points of view were not considered for the purpose of this narrative review.

A first check of the current literature was performed by M.M.V. and V.G., with a consensus subsequently formed by M.N., S.A., and L.C. Supervision of the overall screening, summary, and writing process was carried out by F.C.

## 5. Telerehabilitation in Patients with Fractures

Patients who suffer fractures can be followed up using telemedicine and telerehabilitation. Several medical literature studies have described the effectiveness of this approach. Telerehabilitation in fracture patients aligns with the findings of a study by Seron et al., which analyzed 53 studies spanning 14 prevalent musculoskeletal conditions. The most prevalent telerehabilitation interventions included therapeutic exercises, functional training, and education. The study revealed that telerehabilitation resulted in reduced hospital visits, decreased hospital costs, and improved functional outcomes [83]. In a multicenter randomized controlled trial, Li et al. investigated the impact of remote telerehabilitation, administered for six months, on a cohort of nearly eight hundred women at risk of osteoporotic fragility fractures. A key criterion for discontinuing the study is the occurrence of a fragility fracture [84].

**Figure 1:**
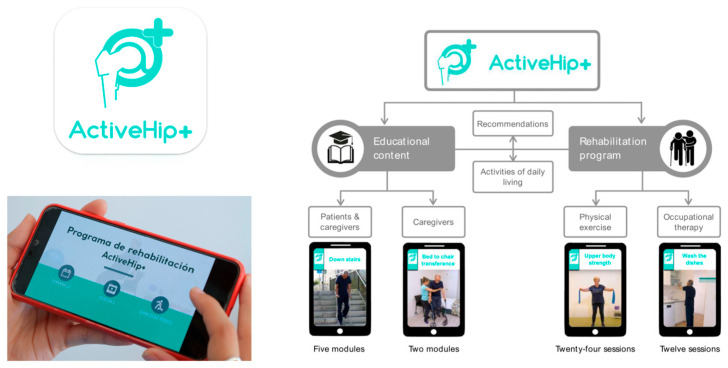
ActiveHip+^®^ is an educational and telerehabilitation program for patients who have undergone surgery after a hip fracture or require hip replacement. Left side of the figure: main features offered in both the health professionals’ environment and the patients’ and caregivers’ environment. Right side of the figure: ActiveHip+^®^ content provided to patients and caregiver. For further details, see the dedicated section in the main text, go to the corresponding article by clicking on the following doi: https://doi.org/10.1002/nur.22218, or, or more information about the App, see the following link: https://play.google.com/store/apps/details?id=nabelia.active_hip (accessed on 20 July 2025). For more information about the project, please also visit the following link: https://www.activehipplus.com/?lang=en (accessed on 20 July 2025).

In the prospective randomized ActiveFLS study, the intervention group included older adults who had sustained a hip fracture. The intervention consisted of a multicomponent approach, including personalized home exercises using an app called @ctive hip^®^ (developer partner: SOCIOEMPRENDE SL, Valencia, Spain), an asynchronous mobile app installed on the patient’s smartphone, for three months. This was followed by nine months of exercises with Vivifrail^®^. (The Vivifrail^®^ project is a program for the Promotion of Physical Exercise that is an international reference for community and hospital intervention for the prevention of frailty and falls in the elderly. Link: https://vivifrail.com/ (accessed on 20 July 2025). The Vivifrail^®^ app is a tool for healthcare professionals and operators to prevent frailty and falls in the elderly. Developer: Miguel Eugenio Izquierdo Redín. Email: mikel.izquierdo@gmail.com. Nation: Spain. Link: https://play.google.com/store/apps/details?id=com.mikelizquierdo.vivifrail&hl=it (accessed on 20 July 2025)) [85]). See Figure 1.

Mora-Traverso et al. implemented an mHealth telerehabilitation and health education program on the physical performance of patients with hip fractures and their family caregivers, based on the ActiveHip+^®^ program. ActiveHip+^®^ is an educational and telerehabilitation program designed for patients who have undergone surgery after a hip fracture or require hip replacement. It also provides specific training and counseling to their caregivers. The program’s primary objective is to enhance the functional recovery of patients who have undergone surgery, thereby promoting their independence in performing daily activities and ultimately improving their overall quality of life [86].

**Figure 2:**
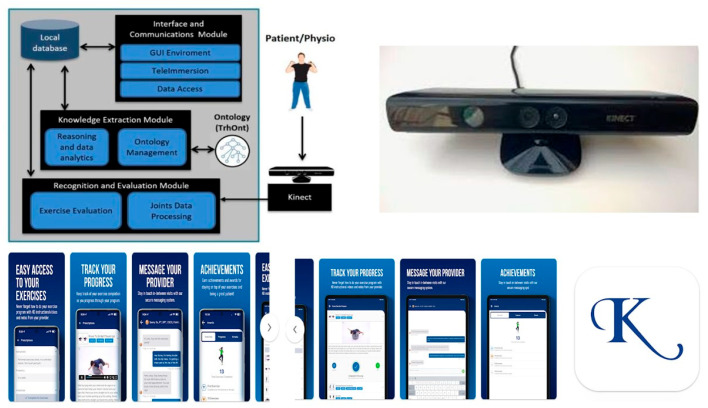
KiReS^®^ (Kinect Telerehabilitation System) is a Kinect-based telerehabilitation system that aims to improve the rehabilitation experience for both physiotherapists and patients. For further details, see the dedicated section in the main text, or go to the corresponding article by clicking on the following link: https://ieeexplore.ieee.org/document/6720717 (accessed on 20 July 2025). At the bottom of the figure are the various screens of the Kriz^®^ physical therapy app (developer: PT Wired Inc.; email: vikram@ptwired.com. Greensboro, NC, USA). Patients can access their personalized home exercise program via mobile device, and using this app, they can access their personalized home exercise program with HD instructional videos. In the “Messages” tab, they can communicate securely with their Kriz^®^ physical therapy provider. For more information about the App, visit the following link: https://play.google.com/store/apps/details?id=com.ptwired.krizpt (accessed on 20 July 2025).

In 2018, Anton D et al. published a research paper on a telemonitoring system specifically designed for rehabilitation. They named it KiReS^®^ (Kinect Telerehabilitation System). KiReS^®^ is a Kinect-based telerehabilitation system that aims to enhance the rehabilitation experience for both physiotherapists and patients. The system offers an intuitive and motivating interface for patients, providing valuable feedback to improve the rehabilitation process. At the same time, it assists physiotherapists in designing, managing, and evaluating physiotherapy protocols and sessions, offering smart data and real-time monitoring. Kinect is an innovative developer kit for spatial processing, equipped with advanced models for computer vision and speech recognition, as well as advanced artificial intelligence sensors. It is a sensor capable of capturing three-dimensional body movements. Based on limb and body movements, specific commands are provided, whether it is a game or a video. KiReS^®^ monitored patients while they performed the exercises prescribed in front of a Kinect device. The system provided a physiotherapist interface that assisted in creating exercises step-by-step. These exercises could be created from scratch or reused if they were already stored in the KiReS database [87] (Figure 2).

In 2019, the results of an Israeli study were published. The study randomized patients over 60 years of age who had undergone surgical hip fractures into three equally sized groups to receive either standard treatment as a control group, conventional rehabilitation treatment with both a patient and a caregiver physically present, or telerehabilitation. Outcome measures include the Functional Independence Measure (FIM) for evaluation of ADL, SF-12 for evaluation of Health-related QOL, the Geriatric Depression Scale (GDS), and The Zarit Caregiver Burden Scale [88].

**Figure 3:**
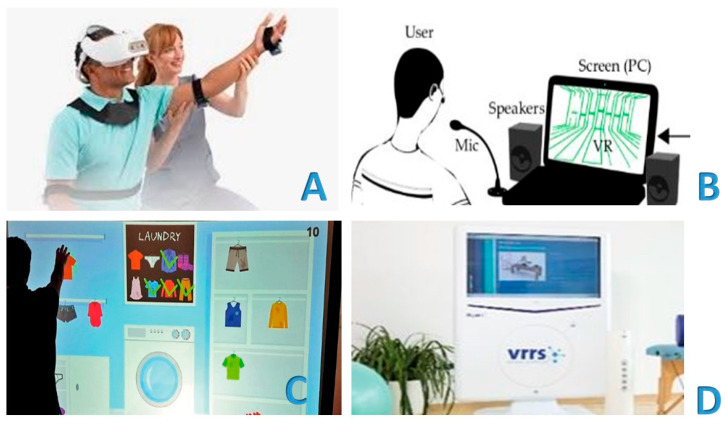
Virtual reality is emerging as a promising tool in telerehabilitation, offering new opportunities for remote rehabilitation. Virtual reality allows for the creation of virtual environments that simulate real or imaginary scenarios, offering the patient the possibility of interacting with them and carrying out specific rehabilitation exercises, even in rehabilitation settings where there is the physical presence of both the patient and the physiatrist and physiotherapist (**A**). Patients can still participate in rehabilitation sessions from home, reducing the need for travel, with virtual reality (**B**). There is also a so-called “non-immersive” virtual reality, which uses monitors or wall projections to produce a 3D image. Therefore, the external environment is not completely eliminated, and the patient receives the impression of a three-dimensional virtual environment, interacting in real time with the representation of their own body (**C**). Figure 3D shows an example of virtual reality. It shows the Khymeia VRRS^®^—Virtual Reality Rehabilitation System—which is an internationally patented Class I certified medical device (**D**). VRRS^®^ (Khymeia Group, Via San Marco 11/C 35129—Padova (PD)—Italy, VAT 03345930287, CCIAA 1998-64134. Email: info@khymeia.com. Link: https://khymeia.com/prodotti/ (accessed on 20 July 2025)) is an advanced, comprehensive, and clinically tested virtual reality system for rehabilitation. It is also characterized by its extreme ease of use, high customization capabilities, and complete automatic reporting. VRRS^®^ is also designed as a “central hub” to which a series of specialized peripheral devices can be connected via a USB, fully synchronized and integrated with the system. VRRS^®^, with its exclusive magnetic kinematic acquisition system, is used as a clinical routine for the rehabilitation of a wide range of pathologies through numerous rehabilitation modules containing the largest library of clinically validated exercises available. The scientific paradigms on which the system is based are, in particular, those of “augmented feedback” and “motor imagery,” principles on which the consolidated experience of promoting motor and functional learning by the central nervous system is based. Augmented feedback, through exercises performed in a virtual environment, allows for the development of “awareness of results” and “awareness of the quality” of the movements performed. In this way, the central nervous system can activate a crucial “physiological learning” mechanism, which requires an increase in movement-specific information to produce a significant improvement in performance quality. For further information, along with the main text, please refer to the publication Pournajaf S, Giovanni Morone G, Goffredo M, Bonaiuti D, Franceschini M. “Virtual reality applied to rehabilitation: clinical evidence and future perspectives,” Italian Journal of Rehabilitation Medicine, Vol. 36, Number 3: 30–42, September 2021 (link: https://springerhealthplus.it/mr/archivio/realta-virtuale-applicata-alla-riabilitazione-evidenze-cliniche-e-prospettive-future/ (accessed on 20 July 2025)) and to the link for the VRRS^®^ device: https://www.fisiomedlambrate.it/featured_post/realta-virtuale/ (accessed on 20 July 2025).

Chirayath et al. recently conducted a study to explore various modalities of managing patients with calcaneal fractures. The authors delved into the technological innovations in management and surgical treatment, as well as the rehabilitation aspects. The study focused on a re-educational program that incorporated virtual reality (VR) technology. VR is emerging as a promising tool in telerehabilitation, offering new opportunities for remote rehabilitation. Patients can access re-educational therapies and receive emotional support directly from home using VR headsets and other tools. VR allows for the creation of virtual environments that simulate real or imaginary scenarios, enabling patients to interact with them and perform specific rehabilitation exercises. This is particularly beneficial in rehabilitation settings where the patient and PRM specialist are physically present. Patients can still participate in re-educational sessions from home, reducing the need for travel. A “non-immersive” VR has also been developed, using monitors or wall projections to produce a 3D image. This type of VR does not completely eliminate the external environment, allowing patients to interact with a three-dimensional virtual environment in real time, with the impression of their own body [89,90,91]. Chirayath et al. integrated VR technology into the program to create a virtual environment tailored to the patient’s needs. This virtual environment encouraged active participation in exercises, potentially improving compliance and motivation during recovery. Additionally, the authors discussed the potential of sensor-based rehabilitation devices and telerehabilitation systems. These systems enable remote assessments and exercise guidance between patients and healthcare professionals [92] (Figure 3).

An equally recent Turkish study employing a method called Home-Based Real-Time Video Conferencing (HBRVC) telerehabilitation investigated a small group of patients with distal radius fractures. The study recruited patients between May 2022 and May 2023, with a specific sample of elderly subjects. All patients underwent volar plating due to a diagnosed and controlled radial fracture. The patients were included in a single-blind randomized study.

The study revealed a dual perspective, with both positive and negative aspects. On the one hand, the HBRVC telerehabilitation program appeared to be as effective as in-person rehabilitation in improving joint range of motion and reducing edema in patients undergoing volar plate fixation for a known fracture. On the other hand, the telerehabilitation method was found to be less effective in improving muscle function and strength compared to in-person rehabilitation [93]. Regarding upper limb fractures, da Silva et al. conducted a systematic review and found that telerehabilitation appears to have favorable effects on functional capacity and pain perception [94].

**Figure 4:**
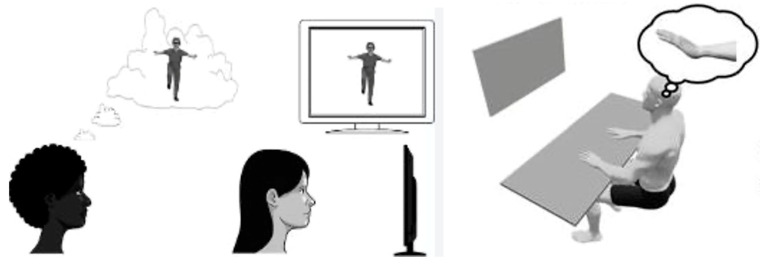
Explanation of the mechanism underlying “motor imagery” used in telerehabilitation technologies. When a subject observes or imagines an action, their mirror neurons activate as if they were performing the action themselves, creating a sort of “internal emulation.” This process can be used to facilitate the recovery of motor functions lost, for example, following a stroke or other types of injury, by leveraging brain plasticity. Regarding the use of “motor imagery” in patients who have suffered a fracture, please refer to the main text.

Kalayci et al. investigated the effects of motor imagery (MI) on distal radius fractures, seeking technological systems and re-educational interventions to enhance treatment effectiveness. MI involves mentally simulating a motor action without physically performing it. For instance, imagining performing a movement without actually moving the involved body part [95]. Techniques that exploit this principle have been used in various fields, such as rehabilitation and sports, to improve motor control and learning both in patients [96,97,98,99] and athletes [100,101]. MI has been shown to improve pain, function, range of motion, and muscle strength in musculoskeletal re-education [102,103], although studies on its effect on upper limb injuries are limited. In the study by Kalayci et al., a group of patients underwent MI in addition to traditional re-education, while another group received only traditional re-education (three times a week for 8 weeks). The study compared the two groups in terms of pain intensity, wrist function, muscle strength, and active range of motion. Statistically significant changes were observed in favor of the MI group in the Patient Rated Wrist Evaluation-function parameter, active range of motion in wrist extension, and hand grip strength. Interestingly, both groups showed improvements in their quality of life [104] (Figure 4).

Among the tools used for telerehabilitation, smartphone-based apps offer the convenience of remotely following re-educational programs, utilizing mobile technologies for patient care and monitoring. Rehabilitation platforms, dedicated software, and apps enable the development of personalized re-educational programs with these supportive features, along with user-friendly training videos. These systems, combined with the intervention of PRM specialists, primary care physicians, and physiotherapists, are user-friendly and facilitate understanding of correct training techniques, even allowing for self-training. Moreover, these systems facilitate data collection and monitoring of patient progress [105,106]. Chen et al. evaluated 31 apps, divided into four categories (smart intervention, angle measurement, smart monitoring, and rehabilitation games). These apps provided both guidance and training for patients’ home rehabilitation (compensating for the high costs and space limitations of traditional rehabilitation methods), as well as methods for evaluating outcomes and actively interacting with users for rehabilitation purposes. Indeed, the smart intervention category had the highest download rate on the app market. Angle measurement tools helped patients with distal radial fractures independently measure their joint angle to assess their rehabilitation status. Many of these apps have achieved good clinical validation over time [107].

Lastly, integrating wearable sensors and software algorithms during telerehabilitation enables the modulation of care for fracture patients, including home monitoring of bone load. Various authors have reported that data from inertial measurement units and pressure insoles in shoes can be fused into machine learning algorithms based on biomechanical data [108,109]. Nurse et al. studied an approach based on this type of technology on patients who had suffered tibial shaft fractures, using a significantly small sample (eight young participants, equally divided between men and women) trying to obtain useful information for remote monitoring of the load on the tibia bone with insoles using a pressure sensor [110].

## 6. Telerehabilitation in Patients with Fractures and Treated with Surgical Interventions

Telerehabilitation programs have also been used and are being studied in patients who have undergone surgical interventions due to fractures. In 2009, Eriksson et al. conducted a small study involving 22 subjects to test the benefits of remote physiotherapy after shoulder surgery. They evaluated clinical outcomes, such as pain (measured using a visual analog scale, VAS) and functional outcomes, including measuring the range of motion [111]: in this study, although with obvious limitations due to the small number of subjects, video monitoring of patients at home had advantages. Since, at least, the non-inferiority of this method had been highlighted at a distance in patients undergoing hip prosthesis for osteoarthritis [112], and since it also seemed to have economic benefits, as in Nelson’s study et al. [113], with data confirming such economic benefits for all orthopedic teleconsultations coming not only from the analysis of the Olsen group in the same year [114] but also from the study of Ohinmaa et al. almost two decades earlier [115], it was decided to extend these studies to the elderly population who had suffered fractures.

During the COVID-19 pandemic, Prieto-Moreno et al. launched a downloadable iPhone app to monitor the rehabilitation of elderly patients undergoing surgery. By 2022, the experience had proven satisfactory (in Spain, 85% app adoption was observed, with a small but significant percentage (64%) of 69 elderly patients recruited using the device), although the authors themselves recommended further data analysis through a specially designed randomized trial [116]. This trial (multicenter in three Spanish hospitals and open-label) was conducted shortly after, with equally valid and positive results, so much so that the App was then used in a greater number of hospitals and not only in Spain but also in Belgium and Portugal [117].

Other experiments aimed to extend the duration of observations. Wu et al. compared the effectiveness of a six-month home-based telerehabilitation program (double the duration of Prieto-Moreno et al. protocol) on a small group of elderly patients with hip fractures treated with total hip replacement. The telerehabilitation group used an Internet-based rehabilitation management system combined with conventional outpatient care. The telerehabilitation group showed a higher functional independence score (functional independence measure, FIM) compared to the control group. Interestingly, the system also allowed for other types of remote monitoring. It was equipped with a Bluetooth connection that enabled the functions of an electronic thermometer, an electronic blood pressure monitor, and an electronic blood glucose monitor by connecting these peripherals to the system [118].

Another study, also Chinese, studied the effects of remote monitoring via smartphone on 31 elderly patients who had suffered a hip fracture [119]. Zhang et al. investigated an internet-based telemedicine tool for remote monitoring and implementing re-educational programs in 29 patients after hip surgery, compared to telephone monitoring. The telerehabilitation management system showed promise in improving the functional recovery of the hip joint and enhancing the ability to perform daily activities and somatic integration to some extent [120].

Pliannuom et al. recently conducted a meta-analysis on PubMed, Embase, Cochrane, ProQuest, and CINAHL up to 3 January 2023. This comprehensive review included 16 studies. The study population consisted of adults aged 60 years and older who had undergone surgery for hip, femoral neck, intertrochanteric, or subtrochanteric fractures and were receiving post-operative care. The researchers assessed various functional outcomes, including the Timed Up and Go (TUG) test, Short Physical Performance Battery (SPPB), and FIM. Notably, these scores improved significantly after home telerehabilitation. Beyond the improvement in functional outcomes, the study also observed a notable increase in the subjects’ daily and instrumental activities of life (ADLs and IADLs). This improvement was accompanied by enhanced compliance with the telerehabilitation protocols. The follow-up modalities allowed for a higher adherence to the protocols, leading to a tendency towards more responsible self-care. Patients demonstrated more careful use and management of therapies, as well as a greater focus on their nutritional status. Among the various delivery methods, phone calls emerged as the most common. This finding raises questions about how these positive results can be achieved using relatively simple means, without necessarily resorting to sophisticated technology [121].

Telerehabilitation platforms are now also integrated with apps that, in addition to video calls, can provide valuable data on the exercises performed and the results achieved, both during each session and at the end of the observation period. This was demonstrated in a randomized trial conducted by a group from Shanghai, a renowned trauma center in China, on patients undergoing total hip replacement. The study compared the traditional physiotherapy approach with telerehabilitation. The traditional physiotherapy group received a standard re-educational intervention in the hospital for one month, followed by outpatient physiotherapy for the next two months. The telerehabilitation group was followed up through a proper mobile platform. Patients were assessed at baseline, four weeks, and twelve weeks after surgery using functional tests (TUG and Berg balance test) and self-administered questionnaires to assess quality of life (QoL), such as the Hip Disability and Osteoarthritis Outcome Score (HOOS) and Short Form 12 (SF-12). While there were no significant differences between the groups, the trial did highlight that the telerehabilitation method was non-inferior to the traditional one [122].

A recent Chinese review highlighted that numerous studies demonstrated the effectiveness of home-based telerehabilitation in promoting the recovery of hip joint function. This approach resulted in improvements in the QoL and, to some extent, psychological factors for elderly patients with hip fractures [123]. This focus on psychological factors and cognition has raised the interest of other authors. Pol et al. conducted a study that explored the benefits offered by telerehabilitation on these aspects and found cognitive positive results [124]. Fernández-González et al. conducted a study to assess the impact of the ActiveHip^®^ application on the families and caregivers of patients with hip fractures. They used two mood assessment scales: the Hospital Anxiety and Depression Scale (HADS) and a functional assessment scale called the Physical Fitness with the International Fitness Scale (IFIS). While no statistically significant differences in care burden were found between the families of patients in the ActiveHip^®^ group and the comparison group, a trend toward lower values was observed in the ActiveHip^®^ group, as well as for anxiety and depression [125].

Mora-Traverso et al. reported similar findings regarding anxiety and depression in patients. The telerehabilitation group showed a greater reduction in the total HADS score and its subscales, including anxiety and depression, compared to the control group. Moreover, the differences between the telerehabilitation group and the control group were consistent at the 3-month follow-up for other self-perceived health indicators [126]. A recent meta-analysis evaluated 17 randomized controlled trials involving over 1500 patients [127]. This analysis separated the functional benefits from the psychological ones, specifically the relief of stress and anxiety. The results regarding the latter were reported with doubts. However, there was a difference in terms of functional benefit regarding the dedicated physical therapy scales. In contrast, a less recent study by Bramanti et al. reported positive aspects of telerehabilitation. Their review analyzed ten studies and found several advantages, including logistical advantages (follow-up of patients living in rural and/or disadvantaged areas) and a very positive psychological impact on the patients [128].

## 7. Discussion

Telemedicine and telerehabilitation emerge as promising tools that can significantly contribute to the management and rehabilitation of patients with fractures, particularly elderly individuals with hip fractures who show adequate functional independence, cognitive status, family or caregiver support, and access to technology. In this specific population, telerehabilitation seems feasible and can deliver multidisciplinary IRPs encompassing physical therapy, occupational therapy, and caregiver education remotely through videoconferencing or digital platforms [129,130]. Evidence from randomized and non-randomized trials support then notion that in selected patients, telerehabilitation yields functional outcomes (e.g., FIM, TUG) that are comparable to or slightly better than conventional home based in person rehabilitation, with high patient and caregiver satisfaction and good adherence [106,129]. QoL and self-efficacy also improve, and caregiver burden is not increased [130,131]. However, systematic reviews and meta-analyses indicate that the effect of telerehabilitation on mobility, adverse events, and pain is uncertain, with no clinically meaningful differences compared to standard care [132]. Telerehabilitation may improve patients’ confidence in ADLs and reduce barriers to access, especially where in-person services are limited. The most suitable telemedicine platforms for elderly patients with hip fractures are those that provide secure, user-friendly videoconferencing, structured exercise modules, and remote monitoring capabilities. The medical literature supports the use of Web-based platforms specifically designed for telerehabilitation, such as the ActiveHip^®^ protocol, which delivers multidisciplinary rehabilitation (physical and occupational therapy, caregiver education) through a dedicated website accessible by patients and caregivers. This approach has demonstrated feasibility and effectiveness in this population, provided there is caregiver support and internet access [129,130].

**Figure 5:**
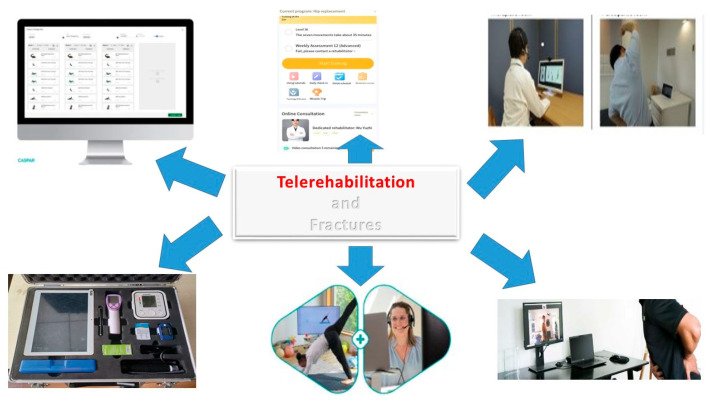
Telerehabilitation uses various technological tools. Smartphone-based rehabilitation offers the possibility of remotely following rehabilitation programs, leveraging mobile technologies for patient care and monitoring. Rehabilitation using platforms, dedicated programs, and apps allows for the development of an individualized training plan with these types of support, along with user-friendly training videos. These, combined with the intervention of the physiatrist, primary care physician, and therapist, are very easy to use and facilitate understanding of correct training techniques, even allowing for self-training. These systems also allow for data collection and monitoring of patient therapy progress, evaluating exercises, and discussing results between the physiatrist, therapist, and patient. For further information, see the main text, the link below (https://apps.apple.com/it/app/caspar-health/id1222630969 (accessed on 20 July 2025)), and the following link: https://play.google.com/store/apps/details?id=com.casparhealth.android.patient&hl=it (accessed on 20 July 2025). Link to the site: https://www.caspar-health.de/en (accessed on 20 July 2025).

Smartphone-based mHealth platforms [e.g., Caspar Health e-system^®^ (Caspar Health is a company of GOREHA GmbH. Location: Neue Schönhauser Str. 20. 10178 Berlin. Email: patientenbetreuung@caspar-clinic.de. Link: https://www.caspar-health.de/en (accessed on 20 July 2025)) and SMPT^®^ (SMPT^®^ is an artificial intelligence (AI)-based platform that digitizes and improves the perioperative process and post-operative patient monitoring visits to control their status. Location: Passeig Bonanova, 10, Sarrià-Sant Gervasi, Barcelona 08022, Spain. Email: Link to the site: https://smtp.health/ (accessed on 20 July 2025))] are tools that offer exercise guidance, real-time feedback, and communication with therapists. These platforms are effective for home-based occupational therapy and physical rehabilitation, with high acceptability and satisfaction among older adults after hip fracture surgery [119,133]. Off-the-shelf videoconferencing applications [e.g., Zoom^®^ (Founder: Eric Yuan; CEO: Eric Yuan (June 2011–); CTO: Xuedong Huang. Founded: 2011, San Jose, CA, USA. Headquarters: San Jose, CA, USA. Link to the Italian site: https://www.zoom.com/it (accessed on 20 July 2025)) and FaceTime^®^ (Developer: Apple; iOS Operating System: macOS, iPadOS. Proprietary License: non-free license. Website: www.apple.com/it (accessed on 20 July 2025))] used on tablets or smartphones, combined with tailored exercise programs and regular therapist follow-up, have been shown to be feasible and acceptable for post-acute telerehabilitation in older adults, including those with fractures [134]. Key features that enhance suitability include intuitive interfaces, clear audiovisual quality, secure data handling, and the ability to involve caregivers in sessions. Platforms should be selected based on patient and caregiver familiarity, local technical support, and integration with existing clinical workflows [130,134] (Figure 5).

The use of telemedicine and telerehabilitation is becoming more prominent in the management of fracture patients treated with surgical interventions, particularly following hip and knee arthroplasty. The medical literature demonstrates that telerehabilitation is generally non-inferior to conventional in-person rehabilitation in terms of functional outcomes, patient-reported outcomes, and safety for patients after total hip or knee arthroplasty, as well as after hip fracture surgery [132,135]. Telerehabilitation can support recovery of mobility, ADLs, and QoL, and may also improve psychological factors such as anxiety and depression in older adults after hip fracture [126,129,136]. Patient, caregiver and operator satisfaction with telerehabilitation is high, and it offers advantages in accessibility, cost, and continuity of care, especially for those with limited access to in-person services [131,137,138]. Telemedicine also facilitates remote monitoring, follow-up, and early identification of complications, which can contribute to patient safety and potentially reduce healthcare utilization [139,140]. However, the evidence for telerehabilitation’s superiority over conventional rehabilitation is limited; most studies show equivalence rather than clear benefit, and some outcomes (e.g., mobility, adverse events) remain uncertain or show only small differences [132,135]. Barriers include technology access, risk of injury without direct supervision, and reduced patient-provider interaction, but these can be mitigated by user-centered program design and clinician support [141].

## 8. Limitations of the Examined Scientific Studies

Several limitations emerged in the scientific medical literature reviewed. Several studies did not examine the long-term outcomes of treatments using various telemedicine methods (e.g., Kalaycı et al.) [104]. Other studies did not assess participant compliance, which is an important measure of whether these methods are not only well accepted by patients but also used effectively. Further studies are certainly needed to explore the potential additional benefits of increasing the number of sessions using a given technology (e.g., for MI). Several studies examined only a few patients (about twenty per comparison group or even fewer, as in the case of Nurse’s work) [110] or a small sample of the study population. For this purpose, studies with a larger sample size could provide more robust information on the effectiveness of a treatment using a specific telerehabilitation protocol. Since these are sometimes not pure clinical studies but rather studies involving experimental technologies and engineering approaches (for example, studies on sensors for load monitoring in patients treated for tibial or calcaneal fractures and subsequently subjected to a rehabilitation program with the concomitant use of insoles or load sensors, as in the case of Nurse’s work) [110], estimating the sample size capable of generating adequate statistical power is particularly complex. In these studies, unlike medical studies, the methodology for calculating the hypotheses a priori and determining the sample size appears to be linked to a greater number of variables, which are sometimes not well-defined or change over the course of the study itself. There are also several practical difficulties in conducting these studies, especially on geriatric patients, who, especially after the stress of hospitalization and ongoing acute and chronic conditions, may lack the strength to participate fully in a study, declining the invitation or compromising the results due to suboptimal compliance [142]. However, one study in this regard suggested that caregiver support can aid in monitoring and even significantly increase adherence to this type of rehabilitation in these patients. The role of caregivers in this setting is further emphasized in a recent study by Ariza-Vega et al. [143] of the same year. Some studies either lack a comparison group or present the two rehabilitation modalities—remote and conventional outpatient—as overlapping and complementary: telerehabilitation is thus presented as a complementary treatment to standard physiotherapy sessions. Many studies therefore explore the benefits of combining telerehabilitation with standard orthopedic and physical therapy care, while superiority studies with significant data are lacking (non-inferiority studies prevail). A greater number of comparison and superiority studies would be desirable.

## 9. Future Perspectives

Current telerehabilitation applications include systems ranging from videophones to (expensive) fully immersive virtual reality systems with haptic interfaces. Naturally, for mobile phones, future prospects are geared towards lowering costs and using increasingly faster data transmission speeds, moving from low-bandwidth to broadband, fiber optics, and, in the future, a mixed model of fiber optics (FTTH), 5G, and satellite internet, with the goal of achieving extremely high speeds and a very high data throughput (with a target of 1 Gbit/s).

Future hopes for virtual reality systems also lie in lower costs. The advent of the European Recovery and Resilience Plan (PNRR) raises the question of the ever-increasing need for digital healthcare data, and this ambitious goal also brings with it challenges that must be addressed in the near future. Several obstacles have been identified to the establishment and advancement of telerehabilitation within the various healthcare systems and the platforms currently in use. There are also professional issues related to the intrinsic practical approach of certain treatments, authorization laws, professional skills development, the disability of the patient to be treated remotely and with which methods, reimbursement, and the scarcity of online assessment and treatment tools and outcome data. These are undoubtedly complex issues that are currently being addressed not only by the healthcare world but also by the commercial and political spheres.

Data is still insufficient to conclusively define whether telerehabilitation is better or worse than traditional rehabilitation. In this regard, while this review aimed to provide an overview of the various technologies and methods used in the rehabilitation field to remotely follow patients, future studies based on clinical experience and with strict methodologies should be focused on in order to assess telerehabilitation’s potential versus conventional rehabilitation.

## 10. Conclusions

Telerehabilitation appears as a feasible, non-inferior, and cost-effective addition or alternative to conventional rehabilitation for patients with fracture. Telerehabilitation seems particularly effective in older adults after hip fractures, improving functional outcomes (e.g., FIM, TUG), ADLs/IADLs, QoL, and adherence when supported by user-friendly platforms and caregiver involvement. These methods appear to offer many advantages when used in frail, older adults who, after a fracture and hospitalization, would have difficulty returning to the clinic for follow-up visits. At the same time, older adults with increased self-sufficiency issues after a fracture and without a strong family network or regular caregivers at home could benefit from remote rehabilitation programs, monitoring themselves and saving on the cost of equipment, travel, and in-person professionals. Superiority over in-person care is still difficult to demonstrate, and effects on pain, mobility, and adverse events remain uncertain. Wider adoption should prioritize secure videoconferencing, structured therapeutic exercise, remote monitoring, and integration into IRPs, while addressing barriers such as digital access, safety without direct supervision, and sustained patient–operator engagement. Future research requires adequately powered, longer-term comparative trials to define optimal candidates, protocols, and technologies (including VR, MI, sensors, and wearables) and to clarify the psychological impacts, safety, and cost-effectiveness across fracture types and settings.

## Figures and Tables

**Table 1:** Benefits of telemedicine and telerehabilitation in non-surgical conditions.

Clinical Area	Intervention Type	Main Outcomes/Benefits
**Diabetes (type 1 and 2)**	Remote monitoring with dedicated software (clinical data exchange, treatment adherence verification, monitoring of blood glucose, HbA1c, ketones)	Improved glycemic control, enhanced treatment adherence, continuous hospital–community support
**Respiratory diseases (including COPD)**	Remote monitoring and supervision within rehabilitation programs	Mixed findings: several studies show no substantial benefit compared with conventional rehabilitation
**Heart failure**	Home telemonitoring and cardiac telemetry (post-surgery and in rehabilitation)	Greater patient comfort, safer follow-up for frail patients, reduced rehospitalization risk; protocols considered safe by cardiology societies
**Oncology (e.g., breast cancer)**	Telemonitoring and dedicated apps for symptom tracking, rehabilitation exercises, and quality of life	Better exercise adherence and symptom monitoring; perceived improvements in quality of life
**Instrumented telemonitoring**	Continuous sensors and remote readings (24 h glucose, malignant arrhythmias, remote spirometry, ECG)	Early and continuous detection of critical parameters and improved follow-up

**Table 2:** Benefits of telemedicine and telerehabilitation in surgical conditions.

Surgical Area	Intervention Type	Main Outcomes/Benefits
**Urology (postoperative)**	Postoperative remote monitoring vs. in-person follow-up	Feasible tele-follow-up with specialist oversight; supports access and monitoring after surgery
**Pelvic floor reconstructive surgery**	Postoperative teleconsultation	High patient satisfaction with teleconsultation for postoperative care
**Gynecologic surgery**	Telemedicine for symptom monitoring (pain, sleep, stress, mental health)	Feasible remote tracking of disabling symptoms; trials also compare virtual vs. in-person visits
**Cardiac surgery (CABG)**	Telemonitoring of symptoms and recovery	Promising recovery to preoperative function by 3–6 months and increased physical activity vs. preoperative levels
**Cardiac rehabilitation (post-cardiac surgery/CVD)**	Telerehabilitation to increase MVPA	Effective at increasing weekly minutes of moderate-to-vigorous physical activity; cardiac rehab is Class IA recommended
**Aortic aneurysm surgery**	Remote telemonitoring of vital signs	No clear improvements in outcomes like anxiety; vital-sign monitoring helpful for early red-flag detection
**Thoracic surgery (lung transplant)**	Telemedicine follow-up/monitoring	Positive impact on postoperative management and monitoring reported
**Thoracic oncology surgery (lung cancer)**	Telemedicine and telerehabilitation	Benefits for remote monitoring and rehabilitation support after surgery

## Abbreviations

Randomized controlled trials: RCTs; chronic obstructive pulmonary disease: COPD; European Society of Cardiology: ESC; Kinect Telerehabilitation System: KiReS; Geriatric Depression Scale: GDS; virtual reality: VR; Home-Based Real-Time Video Conferencing telerehabilitation: HBRVC telerehabilitation; information technology: IT; inertial measurement units: IMUs; Hip Disability and Osteoarthritis Outcome Score: HOOS; Short Form 12: SF-12; quality of life: QOL; computerized tomography: CT; Visual Analogue Scale: VAS; range of motion: ROM; total hip replacement: THR; functional independence measure: FIM; Timed Up and Go test: TUG test; Short Physical Performance Battery: SPPB; Activities of Daily Living: ADL; Instrumental Activities of Daily Living: IADL; Hospital Anxiety and Depression Scale: HADS; Physical Fitness with the International Fitness Scale: IFIS; motor imagery: MI; Evidence-Based Medicine: EBM; artificial intelligence: AI; fiber optics: FTTH; National Recovery and Resilience Plan: NRRP (in Italian, Piano di Ripresa e Resilienza: PNRR).
